# Is Yangxue Qingnao Granule Combined with Antihypertensive Drugs, a New Integrative Medicine Therapy, More Effective Than Antihypertensive Therapy Alone in Treating Essential Hypertension?

**DOI:** 10.1155/2013/540613

**Published:** 2013-02-21

**Authors:** Jie Wang, Xiaochen Yang, Bo Feng, Weidong Qian, Zhuyuan Fang, Wei Liu, Haixia Li, Xiaoke Li, Fuyong Chu, Xingjiang Xiong

**Affiliations:** ^1^Department of Cardiology, Guang′anmen Hospital, China Academy of Chinese Medical Sciences, Beixiange Street No. 5, Xicheng, Beijing 100053, China; ^2^The First Clinical Medical College, Nanjing University of Chinese Medicine, Jiangsu 210029, China; ^3^Department of Cardiology, Traditional Chinese Medicine Hospital of Wujin District, Changzhou 213100, China; ^4^Department of Cardiology, Jiangsu Traditional Chinese Medicine Hospital, Jiangsu 210029, China; ^5^Basic Medical College, Beijing University of Chinese Medicine, Beijing 100029, China; ^6^Department of Cardiology, Beijing Traditional Chinese Medicine Hospital, Capital Medical University, Beijing 100010, China

## Abstract

*Background*. Yangxue Qingnao granule (YQG) combined with antihypertensive drugs, a new integrative medicine therapy, has been widely used for essential hypertension (EH) in China. This study aims to assess the current clinical evidence of YQG combined with antihypertensive drugs for EH. *Methods*. Randomized controlled trials(RCTs) published between 1996 and 2012 on YQG combined with antihypertensive drugs versus antihypertensive drugs in treating EH were retrieved from six major electronic databases, including The Cochrane Library, PubMed, Chinese National Knowledge Infrastructure, Chinese Scientific Journal Database, Chinese Biomedical Literature Database, and Wanfang Data. Meta-analysis was performed on the overall effects on blood pressure. *Results*. Twelve randomized trials were included. Methodological quality of the trials was evaluated as generally low. Meta-analysis showed that YQG combined with antihypertensive drugs demonstrated potential effect for lowing either SBP (MD: −7.31 [−11.75, −2.87]; *P* = 0.001) or DBP (MD: −5.21 [−8.19, −2.24]; *P* = 0.0006) compared to antihypertensive drugs alone. *Conclusions*. It indicated that YQG combined with antihypertensive drugs is more effective than antihypertensive drugs alone in treating EH. However, more RCTs of larger scale, multicentre/country, longer follow-up periods, and higher quality are required to verify the efficacy of integrative medicine therapy over all antihypertensive therapies.

## 1. Introduction

Hypertension is one of the most important modifiable cardiovascular risk factors, contributing to half of the coronary heart disease and almost two-thirds of the cerebrovascular disease burdens [1]. Among them, approximately 90% to 95% of hypertension, affecting more than 1 billion adults worldwide, is the essential hypertension subtype [2, 3]. There is a robust evidence from randomized trials showing that the treatment of hypertension is remarkably effective, and a small reduction in blood pressure (BP) may result in a large reduction in the risk of stroke and myocardial infarction [4]. Although great prevention efforts have been made, the absolute numbers of hypertensive patients are increasing, given the large population and the increasing prevalence of hypertension in developing countries [5]. Chung et al. [6] estimated that the prevalence of hypertension and related cardiovascular morbidity and mortality has increased dramatically in the past 30 years throughout Asia, placing a considerable and growing economic and social burden on countries throughout the region. The hypertensive patients continue to progress to hypertension-related cardiovascular mortality. Thus, more than two-thirds of elderly patients with hypertension worldwide resort to various kinds of complementary or alternative medicine [7]. 

In China, the prevalence of hypertension increased from 7.8% in 1980 to 27.2% in 2001. Hypertension-related cardiovascular mortality in China is predicted to cost $6–9 million per year until 2030. And the economic impact will be particularly pronounced as a high proportion of deaths will occur in people of working age [6]. Fortunately, there is one important characteristic of China's national medical system, that is, traditional Chinese medicine (TCM) and western medicine (WM) complement and cooperate with each other (also known as integrative medicine), being responsible for the health care of Chinese people together [8, 9]. Integrative medicine (IM), therefore, combines the latest scientific advances with the most profound insights of traditional healing systems to regain and preserve health and to assist the patient's own capacities to recover from illness [10]. In the last decades, an increasing popularity of IM has been gained for the treatment of chronic and acute states of illnesses in in-patient treatment [11]. Until now, a variety of high-quality clinical trials in IM have been conducted and published. There are more and more evidence of safety and effectiveness in IM [12]. As we know that TCM has played an important role in the medical care of patients with hypertension-related signs and symptoms for thousands of years in China [13–15]. Modern researches found out that, compared to antihypertensive drugs alone, IM has better efficacy both in blood pressure (BP) and clinical symptoms such as headache, neck and shoulder pain, dizziness, and fatigue [16, 17]. There is no doubt that, in modern time, IM therapy will be widely used for hypertension treatment both in China and other countries [16–20].

Yangxue Qingnao granule (YQG) is a popular Chinese traditional patent medicine (CTPM) for the treatment of essential hypertension (EH), which has been authoritatively recommended by Newly Edited National Chinese Traditional Patent Medicines [21] and reasonable application manual of traditional Chinese patent medicine in internal medicine [22]. YQG contains eleven commonly used herbs, including angelica sinensis, *Ligusticum chuanxiong* Hort, white peony root, prepared radix rehmanniae, uncaria, *Spatholobus* suberectus *Dunn*, *Prunella vulgaris*, cassia seed, pearl shell, rhizoma corydalis, and asarum herb. The mechanism of the prescription maybe related to nourish blood, calm liver, and activate blood circulation according to the theory of TCM [21, 22]. It has been widely used to treat hypertension-related symptoms in clinical practice in China. The most common symptoms include headache, dizziness, giddiness, irritability, and insomnia, which belong to the liver yang hyperactivity and blood deficiency syndrome [21, 22]. Recently, more and more researches demonstrated that YQG could contribute to lowing BP with few side effects both in vitro and in vivo when tested alone [23–28]. Biochemically, BBTD also showed good effect in improving plasma levels of endothelin (ET), calcitonin gene-related peptide (CGRP), and nitric oxide (NO), regulating rennin-angiotensin system (RAS) and decreasing serum levels of urea nitrogen, uric acid, and creatinine [26–28]. 

Currently, YQG combined with antihypertensive drugs, a new integrative medicine therapy, has been widely used as an alternative and effective method for EH in China. And, until now, a large number of randomized controlled trials (RCTs) and case series have been published in Chinese language but have not been evaluated according to the PRISMA systematic review standard. This study aims to assess the current clinical evidence of YQG combined with antihypertensive drugs for EH. 

## 2. Methods

### 2.1. Database and Search Strategies

The literature searches were conducted in The Cochrane Library (October, 2012), PubMed, Chinese National Knowledge Infrastructure (CNKI), Chinese Scientific Journal Database (VIP), Chinese Biomedical Literature Database (CBM), and Wanfang data. We also searched the reference list of retrieved papers. Four main databases in Chinese were searched to retrieve the maximum possible number of trials of YQG for EH because YQG is mainly used and researched in China. All of the searches ended on October 20, 2012. Ongoing registered clinical trials were searched in the website of Chinese clinical trial registry (http://www.chictr.org/) and international clinical trial registry by U.S. national institutes of health (http://clinicaltrials.gov/). The following search terms were used individually or combined: “hypertension”, “essential hypertension”, “Yangxue Qingnao granule”, “nourishing the blood and clearing brain granule”, “clinical trial”, and “randomized controlled trial.” The bibliographies of included studies were searched for additional references. 

### 2.2. Inclusion Criteria

 All the randomized controlled trials (RCTs) based on YQG combined with antihypertensive drugs compared with antihypertensive drugs in patients with essential hypertension were included. There were no restrictions on population characteristics, language, and publication type. The main outcome measure was blood pressure. Duplicated publications reporting the same groups of participants were excluded.

### 2.3. Data Extraction and Quality Assessment

Two authors conducted the literature searching (Xiong, Chu), study selection (Xiong, Yang), and data extraction (Xiong, Qian) independently. The extracted data included authors, title of study, year of publication, study size, age and sex of the participants, diagnosis standard, details of methodological information, treatment process, courses, details of the control interventions, outcomes, and adverse effects for each study. Disagreements were resolved by discussion and reached consensus through a third party (Wang). The methodological quality of trials was assessed independently using criteria from the *Cochrane Handbook for Systematic Review of Interventions*, Version 5.1.0 (Xiong, Yang) [29]. The seven items included random sequence generation (selection bias), allocation concealment (selection bias), blinding of participants and personnel (performance bias), blinding of outcome assessment (detection bias), incomplete outcome data (attrition bias), selective reporting (reporting bias), and other bias. The quality of all the included trials was categorized to low/unclear/high risk of bias (“yes” for a low of bias, “no” for a high risk of bias, and “unclear” otherwise). Then, trials were categorized into three levels: low risk of bias (all the items were in low risk of bias), high risk of bias (at least one item was in high risk of bias), unclear risk of bias (at least one item was unclear). 

### 2.4. Data Synthesis

RevMan 5.1 software provided by the Cochrane Collaboration was used for data analyses. Continuous outcome will be presented as mean difference (MD) and its 95% CI. The statistical heterogeneity was presented as significant when *I* square (*I* ^2^) is over 50% or *P* < 0.1. Fixed effects model was used if there is no significant heterogeneity of the data; random effects model was used if significant heterogeneity existed (*I* ^2^ > 50%). Publication bias would be explored by funnel plot analysis if sufficient studies were found.

## 3. Result

### 3.1. Description of Included Trials

**Figure 1:**
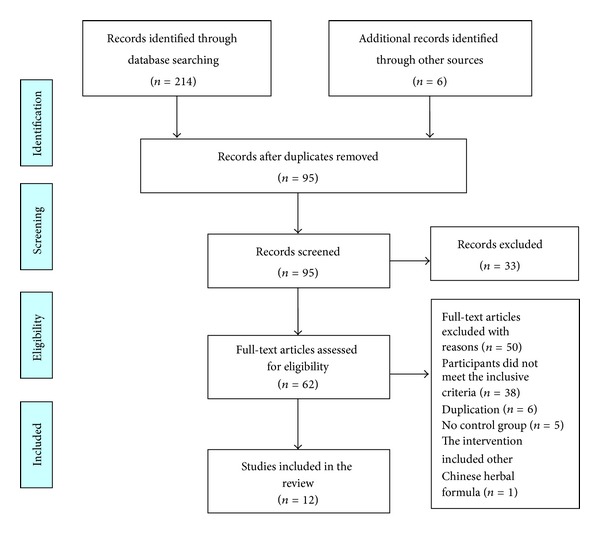
PRISMA 2009 flow diagram.

We identified 220 potentially relevant articles from electronic and manual searches in the six databases. Twelve RCTs [30–41] were included. All the RCTs were conducted in China and published in Chinese. The screening process is summarized in a flow diagram (Figure 1). The characteristics of included trials were summarized in Table 1. 

A total of 1985 patients with essential hypertension were included. Twelve trials specified two diagnostic criteria of essential hypertension, three trials [32–34] used Chinese Guidelines for the Management of Hypertension-2005 (CGMH-2005), three trials [35, 38, 40] used 1999 WHO-ISH guidelines for the management of hypertension (1999 WHO-ISH GMH), and the other six trials [30, 31, 36, 37, 39, 41] only demonstrated patients with essential hypertension without detailed information. Only two trials [35, 38] specified the diagnostic criteria of liver-kidney yin deficiency syndrome and blood stasis syndrome by Guidelines of Clinical Research of New Drugs of Traditional Chinese Medicine (GCRNDTCM) and Diagnostic Criteria of Blood Stasis Syndrome (DCBSY). And the rest ten trials [30–34, 36, 37, 39–41] did not report any TCM diagnostic criteria. 

The interventions of all the trials [30–41] included YQG combined with antihypertensive drugs as shown in Table 1. The controls included antihypertensive drugs alone. The total treatment course duration ranged from 2 to 12 weeks. All of the sixteen trials used the BP as the outcome measure. Adverse effect was described in details. 

### 3.2. Methodological Quality of Included Trials

According to the criteria introduced above, no trial was evaluated as having a low risk of bias. The majority of the included trials were assessed to be of general poor methodological quality. Only one trial of the twelve trials reported the method to generate the allocation sequence (random number table) in the paper [37]. We have tried to contact the authors for further information; however, no detailed information could be get. Therefore, it could not judge whether or not it was conducted properly because of the insufficient information provided. Allocation concealment, blinding of participants and personnel, and blinding of outcome assessment were not mentioned in all trials. No trials reported dropout or withdraw. None of trials had a pretrial estimation of sample size. Only two trials [34, 36] mentioned followup. The results of the assessment of risk of bias are presented in Table 2.

### 3.3. Effect of the Interventions

All the included trials [30–41] compared YQG combined with antihypertensive drugs with antihypertensive drugs alone. A change in blood pressure was reported in all the RCTs. Among them, six trials [33, 34, 38–41] used three classes to evaluate treatment effects, including significantly effective, effective, and ineffective, according to the changes of BP data in GCRNDTCM. Thus, only the rest six trials could be considered for further meta-analysis [30–32, 35–37].

#### 3.3.1. Systolic Blood Pressure (SBP)

The six independent trials [30–32, 35–37] did not show homogeneity in the trial results, chi-square = 130.49, (*P* < 0.00001); *I* ^2^ = 96%. Thus, random-effects model should be used for statistical analysis. The meta-analysis showed there are significant beneficial effect on the combination group compare to the antihypertensive drugs using alone (MD: −7.31 [−11.75, −2.87]; *P* = 0.001) (Table 3). 

#### 3.3.2. Diastolic Blood Pressure (DBP)

The six independent trials [30–32, 35–37] did not show homogeneity in the trial results, chi-square = 141.51, (*P* < 0.00001); *I* ^2^ = 96%. Thus, random-effects model should be used for statistical analysis. The meta-analysis showed there are significant beneficial effect on the combination group compare to the antihypertensive drugs using alone (MD: −5.21 [−8.19, −2.24]; *P* = 0.0006) (Table 4). 

### 3.4. Publication Bias

The number of trials was too small to conduct any sufficient additional analysis of publication bias. 

### 3.5. Adverse Effect

Eight out of twelve trials mentioned the adverse effect [30–33, 35, 36, 38, 41]. Among them, no adverse events were found in three trials [30, 36, 38]. Four trials reported five specific symptoms including dry cough, nausea, epigastric fullness, sore throat, and constipation [31, 32, 35, 41]. One trial reported adverse effect in valsartan group including paroxysmal headache and dizziness [33]. One trial mentioned hypokalemia in indapamide group which may be the adverse effect of indapamide [35]. One trial mentioned dizziness and fatigue in enalapril group [41]. All of the adverse events were not serious. 

## 4. Discussion

This systematic review included twelve randomized trials and a total of 1985 participants. The main findings of this systematic review were that YQG combined with antihypertensive drugs demonstrated potential effect for lowing either SBP (MD: −7.31 [−11.75, −2.87]; *P* = 0.001) or DBP (MD: −5.21 [−8.19, −2.24]; *P* = 0.0006) compared to antihypertensive drugs alone. YQG is an effective adjunctive treatment to antihypertensive drugs in patients with hypertension. However, the evidence remains weak due to poor methodological quality of including studies. Thus, available data are not adequate to draw a definite conclusion of combination therapy for essential hypertension. And the positive findings should be interpreted conservatively due to the following facts. 

Firstly, the methodology of this systematic review is generally low. (1) Randomization: all the included trials claimed randomization; however, only one trial demonstrated on the generation of random allocation [37]. The other trials did not provide sufficient information on randomization and only mentioned that “patients were randomized into two groups.” So, we could not rule out the possibility that the claimed randomization may not be real actually. It could lead to selection bias. (2) Blinding: all of the studies were lack of any blinding method, either blinding of participants and personnel or blinding of outcome assessment. There were six trials [33, 34, 36, 37, 39, 40] conducted by only one author, and three trials [32, 35, 38] conducted by only two authors. It is difficult to accomplish an RCT such as randomization, allocation concealment, blinding and analysis only by one or two doctors. It could lead to performance bias. (3) Analysis of data: only two trials have reported the dropouts, but without the intention-to-treat analysis [31, 36]. Therefore, the positive findings should be interpreted conservatively. It could lead to attrition bias. (4) Placebo controlled: none of the included trials have placebo control. All of them used “A + B versus B” design where patients were randomized to receive YQG plus antihypertensive drugs treatment versus antihypertensive drugs control treatment without a rigorous control for placebo effect. Thus, positive conclusions would be made due to nonspecific placebo effects [42]. (5) Sample size: none of trials had a pretrial estimation of sample size, which indicated the lack of statistical power to ensure appropriate estimation of the therapeutic effect. And all of the included trials were of single center. Sample size calculation should be conducted before enrollment. It is well known that, if methodologically poorly designed, all the trials would show larger differences between experimental and control groups than those conducted rigorously [43–45].

Secondly, another major limitation was the publication bias. All of the trials were conducted in China and published in Chinese. Almost all the RCTs claimed that the positive effect of YQG combined with antihypertensive drugs is better than antihypertensive drugs alone. Negative findings almost have not been reported. We tried to conduct extensive searches for unpublished material, but no unpublished “negative” studies were found. 

Thirdly, syndrome (also known as “pattern” or “zheng”) is the basic unit and key concept in TCM theory, which has been used in China for over 3,000 years. The Chinese herbs and formulas should match the type of syndrome differentiation, which is the basic rule in TCM clinical practice. In this paper, only two trials reported TCM diagnostic criteria with liver-kidney yin deficiency syndrome and blood stasis syndrome [35, 38]. Thus, the trial assessed the clinical effect of combination therapy with positive findings. The rest ten trials have not mentioned any TCM diagnostic criteria at all [30–34, 36, 37, 39–41]. Six trials reported good effect on improving symptoms such as headache, dizziness, and insomnia, which were common symptoms in hypertensive patients. Chinese medicine practitioners believed that treating patients without syndrome differentiation will impair the advantages of Chinese herbs [46–49]. Therefore, the process of syndrome differentiation should be explained clearly and assessed rigorously.

Fourthly, with the increasing awareness of self-care, natural plants as raw materials are favored by people all over the world for their advantages in preventing and curing diseases. However, the safety problem of Chinese herbal medicines is generally concerned [50]. What is more is, as that integrative medicine therapy with both conventional western medicine and tradition medicine becomes the new trend in current medical care, the combined applications of herbs and drugs are increasing. And the potential of interactions between them causes more and more attention worldwide [51–55]. Herb-drug interaction has hence become an important focus of this systematic review. As most of the trials did not reported adverse events of combination therapy strictly, the safety of YQG combined with antihypertensive drugs needs to be monitored rigorously and reported appropriately in the future clinical trials.

In conclusion, there is some encouraging evidence of YQG combined with antihypertensive drugs for lowering BP, but the evidence remains weak due to the poor methodological quality of including studies. More randomized trials with well design and adequate sample size are warranted to support or refute the positive findings in future [56]. In addition, all clinical trials must be carried out and reported according to the CONSORT Statement [42, 57].

## Figures and Tables

**Table 1:** Characteristics and methodological quality of included studies.

Study ID	Sample	Diagnosis standard	Intervention	Control	Course (week)	Outcome measure
Liu et al., 2009 [30]	118	Hypertension diagnostic criteria (unclear)	YQG 4 g tid + control	Valsartan 80 mg qd	4	BP; adverse effect
Chen et al., 2006 [31]	130	Hypertension diagnostic criteria (unclear)	YQG 4 g tid + control	Antihypertensive drugs (no detailed information)	8	BP; adverse effect
Ji and Han, 2011 [32]	160	1999 WHO-ISH GMH	YQG 4 g tid + control	Antihypertensive drugs (no detailed information)	12	BP; adverse effect
Yuan, 2006 [33]	103	1999 WHO-ISH GMH	YQG 4 g tid + control	Valsartan 80 mg qd	2	BP; adverse effect
Shi, 2011 [34]	80	1999 WHO-ISH GMH	YQG 4 g tid + control	Felodipine 5 mg qd	4	BP
Li and Wan, 2006 [35]	80	CGMH-2005; GCRNDTCM; DCBSY	YQG 4 g tid + control	Indapamide 1.25 mg qd	3	BP; adverse effect
Liang, 2011 [36]	100	Hypertension diagnostic criteria (unclear)	YQG 4 g tid + control	Antihypertensive drugs (no detailed information)	8	BP; adverse effect
Fu and Xiao, 2011 [37]	992	Hypertension diagnostic criteria (unclear)	YQG 4 g tid + control	Antihypertensive drugs (no detailed information)	4	BP
Yang et al., 2008 [38]	122	CGMH-2005; GCRNDTCM	YQG 4 g tid + control	Antihypertensive drugs (no detailed information)	4	BP; adverse effect
Lin and Zhou, 2004 [39]	100	Hypertension diagnostic criteria (unclear)	YQG 4 g tid + control	Antihypertensive drugs (no detailed information)	2	BP
Ai, 2012 [40]	102	CGMH-2005	YQG 4 g tid + control	Antihypertensive drugs (no detailed information)	6	BP
Qin, 2008 [41]	68	Hypertension diagnostic criteria (unclear)	YQG 4 g tid + control	Enalapril 10 mg qd	4	BP; adverse effect

**Table 2:** Quality assessment of included randomized controlled trials.

Included trials	Random sequence generation	Allocation concealment	Blinding of participants and personnel	Blinding of outcome assessment	Incomplete outcome data	Selective reporting	Other sources of bias	Risk of bias
Liu et al., 2009 [30]	Unclear	Unclear	Unclear	Unclear	Yes	No	Unclear	High
Chen et al., 2006 [31]	Unclear	Unclear	Unclear	Unclear	Yes	No	Unclear	High
Ji and Han, 2011 [32]	Unclear	Unclear	Unclear	Unclear	Yes	No	Unclear	High
Yuan, 2006 [33]	Unclear	Unclear	Unclear	Unclear	Yes	No	Unclear	High
Shi, 2011 [34]	Unclear	Unclear	Unclear	Unclear	Yes	Yes	Unclear	High
Li and Wan, 2006 [35]	Unclear	Unclear	Unclear	Unclear	Yes	No	Unclear	High
Liang, 2011 [36]	Unclear	Unclear	Unclear	Unclear	Yes	No	Unclear	High
Fu and Xiao, 2011 [37]	Table of random number	Unclear	Unclear	Unclear	Yes	Yes	Unclear	Unclear
Yang et al., 2008 [38]	Unclear	Unclear	Unclear	Unclear	Yes	No	Unclear	High
Lin and Zhou, 2004 [39]	Unclear	Unclear	Unclear	Unclear	Yes	Yes	Unclear	High
Ai, 2012 [40]	Unclear	Unclear	Unclear	Unclear	Yes	Yes	Unclear	High
Qin, 2008 [41]	Unclear	Unclear	Unclear	Unclear	Yes	No	Unclear	High

**Table 3:** Analyses of systolic blood pressure.

Trials		MD [95% CI]	*P* Value
YQG plus valsartan versus valsartan	1	−7.30 [−10.25, −4.35]	<0.00001
YQG plus antihypertensive drugs versus antihypertensive drugs	1	−9.00 [−10.45, −7.55]	<0.00001
YQG plus antihypertensive drugs versus antihypertensive drugs	1	−4.90 [−7.10, −2.70]	<0.0001
YQG plus indapamide versus indapamide	1	−4.38 [−8.28, −0.48]	0.03
YQG plus antihypertensive drugs versus antihypertensive drugs	1	−16.00 [−17.66, −14.34]	<0.00001
YQG plus antihypertensive drugs versus antihypertensive drugs	1	−1.85 [−4.00, 0.30]	0.09
Meta-analysis	6	−7.31 [−11.75, −2.87]	0.001

**Table 4:** Analyses of diastolic blood pressure.

Trials		MD [95% CI]	*P* Value
YQG plus valsartan versus valsartan	1	−11.70 [−13.70, −9.70]	<0.00001
YQG plus antihypertensive drugs versus antihypertensive drugs	1	−8.00 [−9.03, −6.97]	<0.00001
YQG plus antihypertensive drugs versus antihypertensive drugs	1	−2.11 [−3.60, −0.62]	0.006
YQG plus indapamide versus indapamide	1	0.57 [−1.39, 2.53]	0.57
YQG plus antihypertensive drugs versus antihypertensive drugs	1	−7.00 [−8.27, −5.73]	<0.00001
YQG plus antihypertensive drugs versus antihypertensive drugs	1	−3.04 [−4.12, −1.96]	<0.00001
Meta-analysis	6	−5.21 [−8.19, −2.24]	0.0006
